# Physical health of autistic girls and women: a scoping review

**DOI:** 10.1186/s13229-020-00380-z

**Published:** 2020-10-27

**Authors:** Caroline Kassee, Stephanie Babinski, Ami Tint, Yona Lunsky, Hilary K. Brown, Stephanie H. Ameis, Peter Szatmari, Meng-Chuan Lai, Gillian Einstein

**Affiliations:** 1 The Margaret and Wallace McCain Centre for Child, Youth and Family Mental Health, Centre for Addiction and Mental Health, 80 Workman Way, Toronto, ON M6J 1H4 Canada; 2 Dalla Lana School of Public Health, University of Toronto, Toronto, Canada; 3 Department of Sociology, University of Toronto, Toronto, Canada; 4 Azrieli Adult Neurodevelopmental Centre, Centre for Addiction and Mental Health, Toronto, Canada; 5 Department of Psychiatry, Faculty of Medicine, University of Toronto, Toronto, Canada; 6 Department of Health and Society, University of Toronto Scarborough, Toronto, Canada; 7 Department of Psychiatry, The Hospital for Sick Children, Toronto, Canada; 8 Department of Psychology, University of Toronto, Toronto, Canada; 9 Autism Research Centre, Department of Psychiatry, University of Cambridge, Cambridge, UK; 10 Department of Psychiatry, National Taiwan University Hospital and College of Medicine, Taipei, Taiwan; 11 Tema Genus, Linköping University, Linköping, Sweden; 12 Rotman Research Institute, Baycrest Hospital, Toronto, Canada

**Keywords:** Autism, Physical health, Sex differences, Gender, Girls, Women, Scoping review

## Abstract

**Background:**

There is a growing recognition of sex and gender influences in autism. Increasingly, studies include comparisons between sexes or genders, but few have focused on clarifying the characteristics of autistic girls’/women’s physical health.

**Methods:**

A scoping review was conducted to determine what is currently known about the physical health of autistic girls/women. We screened 1112 unique articles, with 40 studies meeting the inclusion criteria. We used a convergent iterative process to synthesize this content into broad thematic areas.

**Results:**

Autistic girls/women experience more overall physical health challenges compared to non-autistic girls/women and to autistic boys/men. Emerging evidence suggests increased prevalence of epilepsy in autistic girls/women compared to non-autistic girls/women and to autistic boys/men. The literature also suggests increased endocrine and reproductive health conditions in autistic girls/women compared to non-autistic girls/women. Findings regarding gastrointestinal, metabolic, nutritional, and immune-related conditions are preliminary and inconsistent.

**Limitations:**

The literature has substantial heterogeneity in how physical health conditions were assessed and reported. Further, our explicit focus on physical health may have constrained the ability to examine interactions between mental and physical health. The widely differing research aims and methodologies make it difficult to reach definitive conclusions. Nevertheless, in keeping with the goals of a scoping review, we were able to identify key themes to guide future research.

**Conclusions:**

The emerging literature suggests that autistic girls/women have heightened rates of physical health challenges compared to non-autistic girls/women and to autistic boys/men. Clinicians should seek to provide holistic care that includes a focus on physical health and develop a women’s health lens when providing clinical care to autistic girls/women.

## Background

Autism spectrum disorder (hereafter autism) is a neurodevelopmental condition characterized by early-onset social communication difficulties and repetitive, stereotyped behaviors. The estimated prevalence rate of autism is approximately 1% worldwide [1], with a higher prevalence among males than females [2, 3]. The widely reported male-to-female ratio for autism prevalence is 4–5:1 but large-scale, population-based epidemiological studies suggest that the ratio is in fact lower, at 3–4:1 [4]. The higher rates of autism among males reflect sex and gender differences in the likelihood of developing autism and potential gender biases in clinical assessment and diagnoses [5, 6].

Autism is highly associated with co-occurring health conditions [7]. It is hypothesized that this likely reflects complex epigenetic and pleiotropic gene–environment interactions and behavioral mechanisms [3, 8], which complicate the clinical presentation of autism. Co-occurring health conditions in autism are associated with varied developmental trajectories [9, 10] and unique social and psychological challenges experienced throughout the life course [3]. In order to improve our knowledge of sex-based and gender-based differences in autism [11], it is critical to better understand the prevalence and characteristics of co-occurring conditions in autistic girls and women.

Most research on co-occurring conditions in autistic girls/women relative to boys/men has focused on psychiatric conditions, suggesting increased internalizing psychopathology in autistic girls/women compared to in boys/men [12–14]. The latest meta-analysis on co-occurring psychiatric diagnoses in autistic people shows that studies with a higher proportion of girls/women tend to find higher rates of depression [15]. However, less attention has been paid to sex and gender differences in autism outside of the domain of mental health, especially regarding physical health. (Note that we define *physical health* in this context to encompass non-mental health conditions within the broad category of medical disorders or problems.) Accurate and in-depth information in this domain, especially concerning autistic girls/women, is essential to the provision of comprehensive and sex- and gender-sensitive health care and is important for elucidating clinically useful subgroups within the autism spectrum. In view of this, we conducted a scoping review of the literature, focused on the extent and range of research pertaining to the physical health of autistic girls/women. Two research questions guided our review: (1) *What do we know about the physical health of autistic girls/women;* and (2) *How specific are these physical health concerns to autistic girls/women, as compared to autistic boys/men, and to non-autistic girls/women?*

## Methods

We conducted a scoping review of the literature following the methodological framework outlined by Arksey and O’Malley [16] and recent Preferred Reporting Items for Systematic Reviews and Meta-analyses standards for scoping reviews (PRISMA-ScR) [17]. Scoping reviews allow a broad survey of the literature in a particular area to determine existing themes and areas of inquiry that are under-researched. Scoping reviews typically do not conduct an assessment of bias in the research nor do they appraise or generate effect sizes [16]. We considered a scoping review to be the most appropriate approach for examining emerging evidence concerning the physical health of autistic girls/women since it was unclear what specific questions should be posed in this area given the limitations of current literature. Therefore, our purposes were to summarize the extent and range of research pertaining to physical health in autistic girls/women and to identify evidence gaps. In this way, we surveyed all of the literature with respect to physical health in autistic girls/women, without restrictions based on study design or comparison groups. Furthermore, as this literature often—unfortunately—conflates gender and sex, it is difficult to tease apart their respective effects. Hence, in interpreting the findings, references to “girls/women” were assumed to refer to biological, cis-gender females and references to “gender” were read very carefully to determine whether they referred to biological sex or gender identity.

We systematically searched the following databases according to PRISMA standards [18]: CINAHL, PubMed, EMBASE, PsycINFO, Scopus, and Web of Science (see Additional file 1: Appendix: Search Strategy). As this was a scoping review aimed at assessing general themes in the published literature rather than analyzing specific types of data, grey literature was not included in the searches. Autism and co-occurring physical health conditions were defined using a combination of keywords and controlled vocabulary applicable to each database (see Additional file 1: Appendix: Search Strategy). We purposely kept the definition of “physical health” as broad as possible, in order to gather a wide range of studies and gain a thorough coverage of the published literature with respect to non-mental health-related conditions. There was no publication type or date restriction at this stage, but the search results were limited to human studies and journal articles written in English. The final database search was performed on December 5, 2019, and references were managed using Mendeley (https://www.mendeley.com/).

A systematic selection process was used to determine the final articles included in this review. After duplicates were removed, two authors (CK and SB) screened titles and abstracts with support from senior authors (M-CL and GE), using broad criteria to allow for the inclusion of any potentially relevant study for further evaluation. Full-text articles were evaluated for inclusion by CK and SB. The pool of studies identified based on screening titles, abstracts, and consultations with senior authors determined the inclusion and exclusion criteria. At this stage, articles were included if they: (1) reported on co-occurring physical health conditions in people with a diagnosis of autism as defined by the DSM-IV, DSM-5, or ICD-10 criteria, or had direct relevance to the physical health of autistic girls/women; (2) included a clearly articulated sex-specific or gender-specific description or analysis of these conditions; (3) studied biological females only, or if the total female autism sample size was ≥ 15 and with at least one-eighth (12.5%) of the total autism sample being biologically female (to ensure that included studies had a sufficient number of girls/women to derive sex-specific or gender-specific information); (4) reported original, English-language research articles or reviews published in peer-reviewed scientific journals; and (5) in the case of review articles, used systematic search methods and included sex-specific or gender-specific analyses and interpretation. Articles were excluded if they were: (1) review articles using non-systematic search methodology; (2) opinion pieces; (3) editorials; (4) case reports; or (5) conference papers. Final decisions on which articles to include were made in discussion within the research team. Articles were grouped by main topic area and study design for organizational clarity. Data were extracted as shown in Tables 1 and 2, with relevant findings summarized in the Results section. We used a convergent iterative process involving multistage revisions among all authors to synthesize included studies into a series of themes that broadly summarize key findings in the literature.

**Table 1:** Overview of included studies (*n* = 40)

Years of publication	Country of origin	Study design
2007–2020	North America = 17 Europe and UK = 12 Asia = 5 Middle East = 3 Australia = 3 Africa = 0 South America = 0	Systematic reviews and meta-analyses = 5 Reviews with systematic search methods = 2 Cross-sectional studies, with population/registry samples = 20 Cross-sectional studies, with clinic/community samples = 13

**Table 2:** Summary of included studies (*n* = 40 unique studies, by themes and then by comparison groups)

Author, country	Study design	Topic area	Sample size	Age (autism sample)	% Female of autism sample	% ID of autism sample	Comparison groups	Key findings
Theme 1: Overall Physical Health Status								
a. Autistic girls/women compared to autistic boys/men								
Rydzewska et al. [19] UK	Cross-sectional registry sample	Prevalence rates for co-occurring health conditions	*N* = 25,063 autism, *N* = 1,523,756 general population controls	0 to 24 years	20.7% (19,880 males, 5183 females)	15.0%	Autistic boys/men	OR autistic girls/women compared to autistic boys/men (reference group): deafness 2.07 [95% CI 2.04–2.10], blindness 2.51 [2.12–2.97], physical disability 2.60 [2.50–2.71]
Rydzewska et al. [20] UK	Cross-sectional registry sample	Prevalence rates for co-occurring health conditions	*N* = 6649 autism, *N* = 3,739,935 general population controls	25 + years	30.7% (4610 males, 2039 females)	29.4%	Autistic men	OR autistic women compared to autistic men (reference group): deafness 1.169 [95% CI 1.001–1.365], blindness 1.232 [1.051–1.443], physical disability 1.504 [1.333–1.697]
Rydzewska et al. [21] UK	Cross-sectional registry sample	Prevalence rates of general health status	*N* = 6649 autism, *N* = 3,739,935 general population controls	25 + years	30.7% (4610 males, 2039 females)	29.4%	Autistic men	Among young adults (25–34 years), autistic women were more likely to have poorer general health compared to autistic men (43.9% autistic women vs. 35.7% autistic men reporting “poor general health”; *χ*^2^ = 13.2, df = 1, *p* < 0.001). No significant sex/gender differences in other age bands
Supekar et al. [22] USA	Cross-sectional registry sample	Prevalence rates for co-occurring health conditions	*N* = 4790 autism, *N* = 1,842,575 general population controls	All ages	Not reported for overall sample	Not reported	Autistic boys/men	Bowel disorders were overall more prevalent in autistic men, but there was a significant higher prevalence in autistic women > 35 years (23% autistic women vs. 10% autistic men, *p* < 0.05)
Davignon et al. [23] USA	Cross-sectional registry sample	Prevalence rates for co-occurring health conditions	*N* = 4123 autism, *N* = 20,615 ADHD, *N* = 2156 diabetes mellitus, *N* = 20,615 general population controls	14 to 25 years	19.3% (3326 males, 797 females)	13%	Autistic boys/men	Rates of various health conditions mostly greater in autistic girls/women compared to autistic boys/men with the largest differences observed for allergy/immunologic conditions, infections, musculoskeletal conditions, neurologic conditions, and psychiatric conditions
Jones et al. [24] USA	Cross-sectional clinic/community sample	Prevalence rates for co-occurring health conditions	*N* = 92 autism, no controls	23.5 to 50.5 years	25% (69 males, 23 females)	*N* = 82 with data, 70% with ID	Autistic men	Autistic women had a median of 16 comorbid medical conditions, whereas autistic men had a median of 10 comorbid medical conditions, *p* = 0.01
Mason et al. [25] UK	Cross-sectional registry sample	Physical quality of life	*N* = 370 autism, no controls	17 to 80 years	42.7% (199 males, 158 females, 13 not reported)	Not reported	Autistic boys/men	Autistic women reported poorer physical quality of life (mean = 45.98, SD = 19.57) than autistic men (mean = 52.98, SD = 17.32)
Fortuna et al. [26] USA	Cross-sectional registry sample	Overall health status	*N* = 255 autism, no controls	18 to 71 years	24.7% (192 males, 63 females)	*N* = 141 with data, 91% with ID	Autistic boys/men	Female sex/gender was associated with lower odds of good or excellent overall health: OR autistic women compared to autistic men (reference group) 0.5 [95% CI 0.2–1.0]
Cashin et al. [27] Australia	Review with systematic search methods	Physical health status	*n* = 6 studies, with samples ranging from *N* = 92 to *N* = 2075 autism	18 + years	Not reported	Not reported	Autistic boys/men	3 studies included sex-specific analyses, with inconsistent findings
Rubenstein et al. [28] USA	Review with systematic search methods	Sex differences in co-occurring conditions	*n* = 69 studies, with samples ranging from *N* = 28 to *N* = 337,000	Not reported	Not reported	Not reported	Autistic boys/men	Insufficient research (hence evidence) to draw conclusions on sex differences in most co-occurring health conditions
b. Autistic girls/women compared to non-autistic girls/women								
Cawthorpe et al. [29] Canada	Cross-sectional registry sample	Prevalence rates for co-occurring health conditions	*N* = 2040 autism, *N* = 761,409 general population controls	All ages	28.6% (1457 males, 583 females)	Not reported	Same-sex general population controls	Autistic girls/women had increased odds compared to non-autistic girls/women for most physical health conditions (encompassing almost all body systems), similar to that in autistic boys/men compared to non-autistic boys/men. Sex differential patterns were also found. (i) Conditions only elevated in autistic girls/women included: blood and blood-forming organ disorders (autistic female OR 1.35 [95% CI 1.11–1.65], autistic male 1.14 [0.96–1.35]), and endocrine, nutritional, metabolic diseases, and immunity disorders (autistic female 1.47 [1.25–1.73], autistic male 0.63 [0.56–0.71]). (ii) Conditions only elevated in autistic boys/men included: complications during mothers’ pregnancy/childbirth (autistic male 1.52 [1.07–2.15], autistic female 0.55 [0.44–0.68]), and genitourinary system diseases (autistic male 1.2 [1.08–1.33], autistic female 0.99 [0.81–1.20])
Croen et al. [30] USA	Cross-sectional registry sample	Prevalence rates for co-occurring health conditions	*N* = 1507 autism, *N* = 15,070 general population matched controls	Adults (mean age 29.0 years, SD 12.2)	26.9% (1102 males, 405 females)	19.2%	Same-sex general population controls	Autistic women had increased odds compared to non-autistic women for most physical health conditions (encompassing almost all body systems), similar to that in autistic men compared to non-autistic men. Sex differential patterns were also found. (i) Conditions only elevated in autistic women included: stroke (autistic female OR 4.97 [99% CI 1.46–16.86], autistic male 1.48 [0.59–3.70]). (ii) Conditions only elevated in autistic men included: autoimmune diseases (autistic male 1.30 [1.01–1.68)], autistic female 1.12 [0.78–1.60]) and gastrointestinal disorders (autistic male 1.50 [1.25–1.79], autistic female 1.05 [0.80–1.39])
Hand et al. [31] USA	Cross-sectional registry sample	Prevalence rates for co-occurring health conditions	*N* = 4685 autism, *N* = 46,850 matched controls	65 years and older	32.2% (3175 males, 1510 females)	43.8%	Same-sex general population controls	Autistic women had increased odds compared to non-autistic women for most physical health conditions (encompassing almost all body systems), similar to that in autistic men compared to non-autistic men. No sex differential patterns were found. The three physical health conditions with the largest ORs in autistic women were epilepsy (OR 20.8 [95% CI 17.7–24.4]), Parkinson’s disease (8.2 [6.2–10.7]), and other gastrointestinal conditions (4.6 [4.1–5.1])
Theme 2: Epilepsy and Related Neurological Conditions								
a. Autistic girls/women compared to autistic boys/men								
Stacy et al. [32] USA	Cross-sectional registry sample	Prevalence rates: including epilepsy	*N* = 913 autism	Weighted mean age 9.91 years (females 10.82 (SD 0.61) vs. males 9.71 (SD 0.27))	18.3% (746 males, 167 females)	Not reported	Autistic boys/men	No significant differences between autistic girls/women and autistic boys/men
Amiet et al. [33] France	Systematic review and meta-analysis	Prevalence rates: pooled risk ratio for epilepsy, and associations with ID	*N* = 1530 autism in sex/gender analyses from 14 studies	All ages	22.2% in sex/gender analyses (1191 males, 339 females)	Not reported	Autistic boys/men	Lower risk for epilepsy in autistic boys/men compared to autistic girls/women (RR 0.55 [95% CI 0.45–0.66], *p* < 0.001; 34.5% in autistic girls/women vs. 18.5% in autistic boys/men)
Ewen et al. [34] USA	Cross-sectional registry sample	Prevalence rates: epilepsy	*N* = 6975 autism	6 to 18 years	18.7% (5671 males, 1304 females)	20.8%	Autistic boys	Higher risk for epilepsy in autistic girls compared to autistic boys in a larger sub-cohort (RR 1.32 [95% CI 1.14–1.52], *p* < 0.05) but no significant findings in a smaller sub-cohort. Independent positive associations between epilepsy and intellectual disability, language impairment, core autism symptom, and motor dysfunction
Viscidi et al. [35] USA	Cross-sectional registry sample	Prevalence rates: epilepsy	*N* = 5815 autism	All ages, majority between 4 and 12 years (~ 75%)	17.5% (4800 males, 1015 females)	*N* = 4894 with data, 15.5%	Autistic boys/men	Epilepsy was more prevalent in autistic girls/women (7.0%) than in autistic boys/men (3.9%, *p* < 0.001). Co-occurring epilepsy in autism was associated with older age, lower cognitive ability, poor adaptive language functioning, developmental regression, and more severe autism symptoms; most of the associations were driven by IQ
Bowers et al. [36] USA	Cross-sectional clinic/community sample	Prevalence rates: epilepsy, in preterm vs. term births	*N* = 883 autism	0 to 18 years	17.6% (728 males, 155 females)	*N* = 853 with data, 34.5%	Autistic boys	Seizure disorders were more frequent among autistic boys born preterm vs. those born term (17.0% vs. 8.5%, *p* = 0.01); no such preterm–term differences were found in autistic girls
Ben-Itzchak et al. [37] Israel	Cross-sectional clinic/community sample	Prevalence rates and sex differences in neurological phenotypes, including epilepsy	*N* = 663 autism	1 to 15 years	13.0% (577 males, 86 females)	35.0%**	Autistic boys	Neurological anomalies were more prevalent in autistic girls than in autistic boys, including microcephaly (15.1% vs. 4.5%, *χ*^2^ = 15.0, *df* = 1, *p* < 0.001) and minor neurological–musculoskeletal deficits (73.8% vs. 57.1%, *χ*^2^ = 8.0, *df* = 1, *p* < 0.001), but no significant sex differences were found for seizures or macrocephaly
*Supekar et al. [22] USA	Cross-sectional registry sample	Prevalence rates for co-occurring health conditions, including epilepsy	*N* = 4790 autism, *N* = 1,842,575 general population controls	All ages	Not reported for overall sample	Not reported	Autistic boys/men	Overall higher prevalence of epilepsy in autistic girls/women (18.54%) than in autistic boys/men (15.14%, *p* < 0.05); this finding was modulated by age, that epilepsy was female-predominant in 0–18 years and 18–35 years, but male-predominant in > 35 years of age
b. Autistic girls/women compared to non-autistic girls/women								
Ingudomnukul et al. [38] UK	Cross-sectional clinic/community sample	Prevalence rates for co-occurring health conditions, including epilepsy	*N* = 54 autism, *N* = 74 mothers of autistic children, and *N* = 183 mothers of typically developing children (controls)	19 to 63 years	100%	Not reported	Same-sex general population controls	Epilepsy rates were higher in autistic women (7.4%) compared to control women (1.1%, *p* < 0.05)
Pohl et al. [39] UK	Cross-sectional clinic/community sample	Prevalence rates for co-occurring health conditions, including epilepsy	*N* = 415 autism, *N* = 415 age-matched controls	Mean age 36.39 years (SD 11.98)	100%	Not reported	Same-sex general population controls	Epilepsy rates were higher in autistic women (4.1%) compared to non-autistic women (1.4%, *p* = 0.016)
*Croen et al. [30] USA	Cross-sectional registry sample	Prevalence rates for co-occurring health conditions, including epilepsy	*N* = 1507 autism, *N* = 15,070 general population matched controls	Adults (mean age 29.0 years, SD 12.2)	26.9% (1102 males, 405 females)	19.2%	Same-sex general population controls	Autistic women had increased odds compared to non-autistic women for epilepsy and recurrent seizures (OR 34.09 [99% CI 18.51–62.79]); a similar but smaller effect was found in autistic men compared to non-autistic men (11.53 [7.74–17.17])
*Hand et al. [31] USA	Cross-sectional registry sample	Prevalence rates for co-occurring health conditions, including epilepsy	*N* = 4685 autism, *N* = 46,850 matched controls	65 years and older	32.2% (3175 males, 1510 females)	43.8%	Same-sex general population controls	Autistic women had increased odds compared to non-autistic women for epilepsy (OR 20.8 [95% CI 17.7–24.4]) and Parkinson’s disease (8.2 [6.2–10.7]); a similar but smaller effect was found in autistic men compared to non-autistic men for epilepsy (18.0 [16.1–20.2])
Theme 3: Endocrine and Reproductive Health Conditions								
a. Studies with no comparison group								
Hamilton et al. [40] USA	Cross-sectional clinic/community sample	Prevalence rates for menstruation complications	*N* = 124 autism	10 to 25 years	100%	Not reported	None	Autistic girls/women commonly experienced dysmenorrhea (91%), premenstrual syndrome (96%), and 33% endorsed autism-associated difficulties during the menstrual cycle (increased irritability/aggression before menses, worsening of autistic behaviors, and increased repetitive movements and obsessive behaviors)
Bitsika and Sharpley [41] Australia	Cross-sectional clinic/community sample	Effects of menarche on sensory features of autism	*N* = 53 autism	6 to 17 years	100%	Not reported	None	Autistic girls who had reached menarche had lower sensation seeking (less sensory interests) (*F*_(25,27)_ = 2.113, *p* = 0.030) and multisensory processing (*F*_(7,45)_ = 3.187, *p* = 0.008) compared to those who had not yet reached menarche
b. Autistic girls/women compared to non-autistic girls/women								
*Croen et al. [30] USA	Cross-sectional registry sample	Prevalence rates for co-occurring health conditions, including endocrine disorders	*N* = 1507 autism, *N* = 15,070 general population matched controls	Adults (mean age 29.0 years, SD 12.2)	26.9% (1102 males, 405 females)	19.2%	Same-sex general population controls	Autistic women had increased odds compared to non-autistic women for thyroid diseases (OR 1.85 [99% CI 1.20–2.85]); a similar but larger effect was found in autistic men compared to non-autistic men (3.34 [2.18–5.11])
*Hand et al. [31] USA	Cross-sectional registry sample	Prevalence rates for co-occurring health conditions, including endocrine and menopausal disorders	*N* = 4685 autism, *N* = 46,850 matched controls	65 years and older	32.2% (3175 males, 1510 females)	43.8%	Same-sex general population controls	Autistic women had increased odds compared to non-autistic women for thyroid disorders (OR 2.5 [95% CI 2.2–2.8]); a similar but larger effect was found in autistic men compared to non-autistic men (3.7 [3.3–4.0]). Autistic women did not differ from non-autistic women on rates of menopausal disorders (1.1 [0.9–1.5])
Steward et al. [42] UK	Cross-sectional clinic/community sample	Autistic women’s experiences with menstruation	*N* = 123 autism, *N* = 114 controls	16 to 60 + years	100%	Not reported	Same-sex general population controls	Autistic women highlighted autism-specific issues during the menstrual cycle, including a cyclical amplification of autism-related challenges, sensory differences, and emotional regulation challenges, which had a significant negative impact on their lives
*Ingudomnukul et al. [38] UK	Cross-sectional clinic/community sample	Prevalence rates for co-occurring health conditions, including female-specific endocrine conditions	*N* = 54 autism, *N* = 74 mothers of autistic children, and *N* = 183 mothers of typically developing children (controls)	19 to 63 years	100%	Not reported	Same-sex general population controls	Autistic women, compared to control women, had higher rates of polycystic ovary syndrome (PCOS, 11.3% vs. 2.7%, *p* < 0.05), delayed puberty (7.4% vs. 0.5%, *p* < 0.01), hirsutism (29.6% vs. 4.4%, *p* < 0.001), irregular menstrual cycle (57.4% vs. 28.6%, *p* < 0.001), unusually painful periods (44.4% vs. 28.0%, *p* < 0.05), and history of severe acne (27.8% vs. 7.1%, *p* < 0.001)
*Pohl et al. [39] UK	Cross-sectional clinic/community sample	Prevalence rates for co-occurring health conditions, including female-specific endocrine conditions	*N* = 415 autism, *N* = 415 age-matched controls	Mean age 36.39 years (SD 11.98)	100%	Not reported	Same-sex general population controls	Autistic women, compared to non-autistic women, had higher rates of irregular menstrual cycle (46.3% vs. 34.0%, *p* = 0.0002), unusually painful periods (39.3% vs. 26.3%, *p* = 0.00004), premenstrual syndrome in contraceptive pill users (24.0% vs. 13.8%, *p* = 0.001), severe acne in non-contraceptive pill users (21.3% vs. 5.9%, *p* = 0.002), precocious puberty (3.1% vs. 0.5%, *p* = 0.003), and early growth spurt (20.2% vs. 12.8%, *p* = 0.002)
Cherskov et al. [43] UK	Cross-sectional registry sample	Prevalence rates for polycystic ovary syndrome (PCOS)	*N* = 971 autism, *N* = 4855 general population controls	Mean age 30.3 years (SD 9.1)	100%	Not reported	Same-sex general population controls	Prevalence of PCOS was higher in autistic women compared to non-autistic women (by Read code, 2.3% vs. 1.1%, *p* < 0.01, OR 2.01 [95% CI 1.22–3.30]; by NIH criteria, 7.4% vs. 3.1%, *p* < 0.001, 2.50 [1.87–3.35]; by Rotterdam criteria, 7.8% vs. 3.5%, *p* < 0.001, 2.33 [1.76–3.08])
Chiang et al. [44] Taiwan	Cross-sectional registry sample	Prevalence rates for cancer, including ovarian cancer	*N* = 8438 autism, *N* = 76,332 general population controls	0 to 25 + years	17.9% (6931 males, 1507 females)	Not reported	Same-sex general population controls	Autistic girls/women had a higher standardized incidence ratio (SIR) for ovarian cancer compared to non-autistic girls/women (SIR 9.21 [95% CI 1.12–33.29])
Sundelin et al. [45] Sweden	Cross-sectional registry sample	Pregnancy outcomes	*N* = 1382 autism (*N* = 2198 births), *N* = 503,846 general population controls (*N* = 877,742 births)	Adults of child-bearing age	100%	Not reported	Same-sex general population controls	Autistic women, compared to non-autistic women, were at increased risks for preeclampsia (OR 1.34 [95% CI 1.08–1.66]), giving preterm birth (1.30 [1.10–1.54]), medically indicated preterm birth (1.41 [1.08–1.82]), and receiving elective cesarean delivery (1.44 [1.25–1.66])
Miscellaneous Emerging Findings (all studies offered information regarding autistic girls/women in comparison with autistic boys/men)								
a. Additional neurological conditions								
Memari et al. [46] Iran	Cross-sectional clinic/community sample	Prevalence rates for co-occurring health conditions	*N* = 91 autism	6 to 14 years	25.3% (68 males, 23 females)	Not reported	Autistic boys	Autistic girls had higher prevalence of neurological conditions (~ 46%) than autistic boys (~ 19%, *p* = 0.02)
Rubenstein et al. [47] USA	Cross-sectional registry sample	Temporal trends in co-occurring health conditions	*N* = 6379 autism	8-year-olds	18.0% (5230 males, 1149 females)	4.3%	Autistic boys	Rates of change of prevalence for neurological conditions over 2002–2010 were the same for autistic boys and autistic girls
Mouridsen et al. [48] Denmark	Cross-sectional registry sample	Prevalence rates for cerebral palsy	*N* = 4180 autism (ICD-10 Asperger’s syndrome)	4 to 31 years	17.9% (3431 males, 749 females)	0%	Autistic boys/men	Increased rates for cerebral palsy in autistic people (0.65%) than in the general population (0.17%), but no difference between autistic girls/women (0.80%) and autistic boys/men (0.61%, *p* = 0.56)
b. Obesity and overweight								
Broder-Fingert et al. [49] USA	Cross-sectional registry sample	Prevalence rates for obesity and overweight	*N* = 2976 autism, *N* = 3696 general population controls	2 to 20 years	20.7% (2359 males, 617 females)	Not reported	Autistic boys/men	Autistic girls/women were less likely to be obese compared to autistic boys/men (OR 0.71 [95% CI 0.55–0.93]), but this did not hold for overweight (1.06 [0.81–1.39])
Garcia-Pastor et al. [50] USA	Cross-sectional clinic/community sample	Prevalence rates for obesity and overweight	*N* = 78 autism	7 to 48 years	28.2% (56 males, 22 females)	Not reported	Autistic boys/men	Overweight and obesity were more prevalent in autistic men than in autistic women (*p* = 0.035); no differences were found between autistic girls and autistic boys
c. Gastrointestinal, metabolic, or nutritional problems								
Yang et al. [51] China	Cross-sectional clinic/community sample	Gastrointestinal symptoms	*N* = 169 autism, *N* = 172 controls	3 to 12 years	14.2% (145 males, 24 females)	Not reported	Autistic boys	Autistic girls had greater likelihood of gastrointestinal problems than autistic boys (OR 3.88 [95% CI 1.33–11.35], *p* = 0.013); more gastrointestinal symptoms were correlated with more severe core autistic symptoms
Tseng et al. [52] Taiwan	Systematic review and meta-analysis	Iron deficiency	*n* = 25 studies, with *N* = 1603 autism (across 3 analyses), *N* = 1507 controls	0 to 18 years	20.0% (no individual numbers)	Not reported	Autistic boys	No sex/gender differences in iron levels in autistic children
Rossignol and Frye [53] USA	Systematic review and meta-analysis	Mitochondrial disease (MD)	*n* = 65 studies, with *N* = 648 autism (among these *N* = 112 also with mitochondrial disease)	0 to 20 years	39% in autism plus MD, 19% in autism only (no individual numbers)	Not reported	Autistic boys/men	Autism–mitochondrial disease group had higher proportion of autistic girls/women (39%) than the autism-only group (19%, *χ*^2^ = 18.7, *p* < 0.0001); autistic boys/men are the majority in both groups
Guo et al. [54] China	Cross-sectional clinic/community sample	Vitamin A and vitamin D deficiencies	*N* = 332 autism, *N* = 197 controls	Mean age 4.87 years (SD 1.53)	13.9% (286 males, 46 females)	Not reported	Autistic boys	Autistic girls had lower serum 25-OH vitamin D than autistic boys (*p* < 0.05)
d. Immune profile								
Masi et al. [55] Australia	Cross-sectional clinic/community sample	Cytokines	*N* = 144 autism	2 to 18 years	21.5% (113 males, 31 females)	Not reported	Autistic boys	In autistic girls, reduced levels of IL-1β, IL-8, MIP-1β, PDGF-BB and VEGF were associated with increased autism symptoms, while in autistic boys this was the case only for reduced PDGF-BB. Cytokine expression was moderated by sex/gender
Hu et al. [56] China	Cross-sectional registry sample	Cytokines	*N* = 87 autism, *N* = 41 controls	1 to 6 years	17.2% (72 males, 15 females)	Not reported	Autistic boys	Overall, autistic children had higher plasma levels of eotaxin, TGF-β1, and TNF-α than non-autistic children. In autistic girls, only the increase in eotaxin was statistically significant, whereas in autistic boys, the most consistent increase was in TGF-β1. Possible differential immune profiles in autistic girls vs. boys
Saghazadeh et al. [57] Iran	Systematic review and meta-analysis	Cytokines	*n* = 38 studies, with *N* = 1393 autism, *N* = 1094 controls	All ages	36 studies with sex/gender info; 1582 males and 446 females in total sample; % of autistic females not reported	Not reported	Autistic boys/men	Overall, autistic individuals had higher concentrations of pro-inflammatory cytokines IFN-γ, IL-1β, IL-6, and TNF-α than controls; meta-regression revealed moderation effects of sex/gender (difference in the percentage of males between autism and control groups) for IL-1β and TNF-α
e. Autism symptoms associated with physical health								
Moseley et al. [58] UK	Systematic review and meta-analysis	Sex differences in autistic symptoms	*N* = 254 autism, *N* = 273 controls	Weighted mean age 36.4 years (females 37.5 (SD 14), males 35.3 (SD 13.4))	53.5% (118 males, 136 females)	0%	Autistic boys/men	Autistic girls/women had more profound sensorimotor symptoms than autistic boys/men (*t*_(252)_ = 4.346, *p* < 0.001)

^*^ Same study listed again but under different themes

^**^ Calculated using mean and standard deviations from IQ scores, for males and females

*CI* confidence interval, *OR* odds ratio, *RR* risk ratio, *SD* standard deviation, *SIR* standardized incidence ratio

## Results

### Search results

**Fig. 1:**
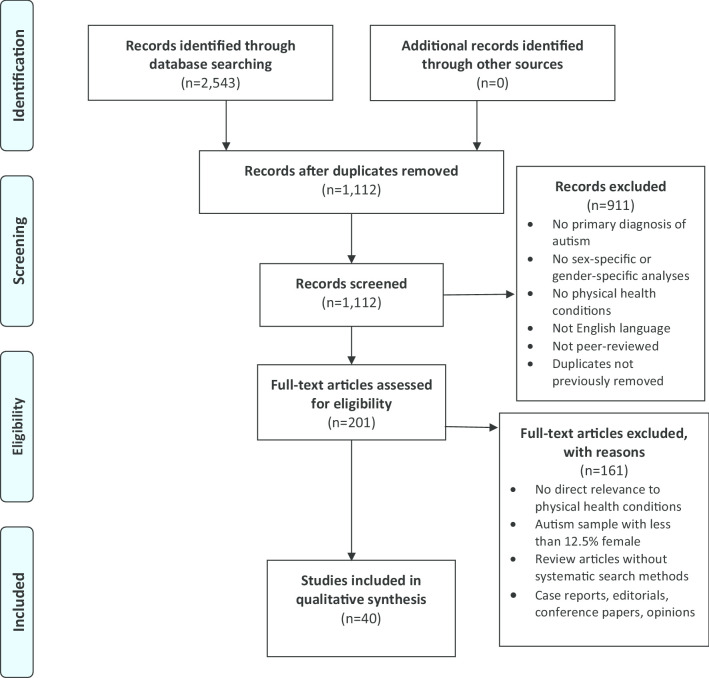
PRISMA flow diagram for study selection

We screened a total of 1112 unique citations and reviewed the full text of 201 articles, with 40 studies ultimately meeting the inclusion criteria (Fig. 1). The majority of the studies were from North America and Europe/UK, cross-sectional, and about general prevalence rates for health conditions in autistic individuals (Table 1). The articles included descriptive studies of autistic girls/women only, as well as studies of autistic individuals of all ages and functional levels that compared autistic girls/women to autistic boys/men and/or non-autistic girls/women (Table 2).

Three major themes on the physical health status of autistic girls/women emerged from the existing literature: (1) *Autistic girls/women experience more overall physical health conditions than autistic boys/men and non-autistic girls/women*; (2) *Epilepsy has been most commonly researched and seems more common in autistic girls/women compared to autistic boys/men and non-autistic girls/women;* and (3) *Autistic girls/women may experience more menstruation-related complications, endocrine and reproductive health conditions compared to non-autistic girls/women*. Finally, studies that met the inclusion criteria but that did not align with the three major themes are described in a fourth section, *Miscellaneous Emerging Findings*. Details of sample characteristics, methodology, and statistics are provided in Table 2.

### Summary of key themes

#### Theme 1: Overall Physical Health Status

Thirteen out of the 40 included studies focused on the overall physical health status of autistic girls/women [19–31], with respect to multiple conditions across body systems.

##### Autistic girls/women compared to autistic boys/men

Six studies explored the prevalence of various co-occurring physical health conditions in autistic girls/women and autistic boys/men [19–24]. Two of these six studies focused on population-based registry data: one in autistic children and youth (25,063 individuals with autism) [19] and the other in autistic adults (6649 with autism) [20]. Both reported higher odds for deafness, blindness, and physical disability in autistic girls/women compared to autistic boys/men [19, 20]. A third study [21], a follow-up on the same adult cohort in [20], found that autistic women in the 25–34 years of age range were more likely to report “poor general health” compared to autistic men [21]. Two additional studies reported on prevalence of physical health conditions as a percentage of their total registry-based autism sample [22, 23]. In 4123 autistic youth in a clinical registry, most physical health conditions were more common in autistic girls/women than in autistic boys/men (e.g., allergy/immunologic conditions, infections, musculoskeletal conditions, neurologic conditions) [23]. In 4790 autistic people of all ages from large medical registry cohorts, there was a higher prevalence of bowel disorders in autistic women compared to autistic men > 35 years of age [22]. The sixth study found a higher median number of medical comorbidities in autistic women compared to autistic men, in a community sample of 92 autistic adults [24].

Two studies further examined sex/gender as a predictor of physical quality of life and overall health status in 370 [25] and 255 [26] autistic adults. Female sex/gender was a significant predictor of lower physical health-related quality of life [25] and lower overall health [26]. Finally, two literature reviews on the physical health status of autistic adults regardless of sex/gender [27] and sex differences in developmental and medical endophenotypes in autism [28], respectively, reported there was insufficient research to reach consensus about sex/gender differences.

##### Autistic girls/women compared to non-autistic girls/women

Three studies with population-based registry samples examined the overall physical health status of autistic girls/women compared to non-autistic girls/women [29–31]. Two of them compared autistic adults to same-sex matched controls (2040 autistic individuals vs. 761,409 general population individuals [29] and 1507 autistic individuals vs. 15,070 general population controls [30]), and the other exclusively studied autistic seniors (≥ 65 years of age) who were enrolled in US-based Medicare (4685 with autism vs. 46,850 controls [31]). All three studies found elevated odds for most physical health conditions in autistic girls/women compared to non-autistic girls/women [29–31]. As a side note (also corresponding to the previous section), two of them also described odds ratios (ORs) of physical health conditions in autistic girls/women (reference to non-autistic girls/women) in contrast to autistic boys/men (reference to non-autistic boys/men) [29, 30]: Some conditions showed elevated ORs only in autistic girls/women, whereas others showed elevated ORs only in autistic boys/men (Table 2), alongside the majority of other conditions showing sex-similar patterns of elevated ORs across autistic individuals [29–31].

#### Theme 2: Epilepsy and Related Neurological Conditions

Eleven out of the 40 included studies specifically focused on, or included, epilepsy when examining sex/gender differences in neurological conditions in autistic individuals [22, 30–39].

##### Autistic girls/women compared to autistic boys/men

Five studies compared autistic girls/women to autistic boys/men with respect to epilepsy [22, 32–35]. While one study reported no sex differences in ORs for epilepsy [32], the other four studies found elevated rates of epilepsy in autistic girls/women compared to autistic boys/men. However, this finding was also associated with age, cognitive ability, adaptive functioning, language ability, and autism symptom severity [22, 33–35]. One meta-analysis with pooled sample sizes of 1530 autistic children and adults from 14 studies [33], and another study with 6975 autistic children from registry-based cohorts [34], found higher rates of epilepsy in autistic girls/women compared to autistic boys/men. They also found positive associations between rates of epilepsy and intellectual disability [33, 34], language impairment, core autism symptoms, and motor dysfunction [34]. Two other registry-based studies on prevalence rates for comorbid health conditions, including epilepsy, in 5815 [35] and 4790 [22] autistic individuals found higher prevalence of epilepsy in autistic girls/women compared to autistic boys/men. The sex difference patterns were modulated by age [22, 35], cognitive ability, adaptive language functioning, developmental regression, and autism symptom severity [35].

In addition, two studies specifically explored neurological profiles including seizure disorders in autistic children from community-based samples [36, 37]. One study of 883 autistic children found seizure disorders to be more frequent among autistic boys born preterm vs. those born term, while no preterm–term differences were found in autistic girls [36]. The other study on 663 autistic children found higher prevalence of neurological anomalies (e.g., microcephaly, minor neurological–musculoskeletal deficits) in autistic girls than in autistic boys, but no sex differences in seizures [37].

##### Autistic girls/women compared to non-autistic girls/women

Four studies reported risks of epilepsy in autistic girls/women against non-autistic girls/women [30, 31, 38, 39]. A population-based registry study of autistic adults found that autistic women were at significantly higher risks of having epilepsy than non-autistic women [30]. In another registry study on physical health in autistic seniors, epilepsy shows the largest OR among all conditions in autistic women compared to non-autistic women [31]. Similarly, two additional community-based studies reported elevated rates of epilepsy in autistic women compared to non-autistic women in a sample of 54 autistic women and 183 non-autistic women [38] and in another sample of 415 autistic women and 415 non-autistic women [39].

#### Theme 3: Endocrine and Reproductive Health Conditions

Ten out of the 40 included studies focused on endocrine and reproductive health conditions in autistic girls/women only [40, 41] or in comparison with non-autistic girls/women [30, 31, 38, 39, 42–45].

##### Studies with no comparison group

Two community-based studies reported menstruation-related health challenges in autistic girls/women [40, 41]. One online survey with 124 autistic girls/women found > 90% of them experienced dysmenorrhea and premenstrual syndrome (PMS), while one-third endorsed increased autism-associated difficulties during the menstrual cycle [40]. The other study on the effects of menarche on the sensory features of autism in 53 autistic girls reported that those who reached menarche had lower sensory interests and multisensory processing than those who had not yet reached menarche [41].

##### Autistic girls/women compared to non-autistic girls/women

Eight studies examined endocrine and reproductive health conditions in autistic girls/women compared to non-autistic girls/women [30, 31, 38, 39, 42–45]. A population-based registry study found that autistic women were at higher risks of having thyroid diseases than non-autistic women [30]. Another registry study on physical health in autistic seniors yielded similar findings regarding thyroid diseases, but autistic women had a comparable rate of menopausal disorders compared to non-autistic women [31]. A community-based qualitative study on 123 autistic and 114 non-autistic women’s experiences of menstruation found that, despite many overlapping challenges reported by both groups, autistic women highlighted amplification of autism-related (e.g., sensory and emotional regulation) challenges in sync with the menstrual cycle, which had significant negative impact on their lives [42]. Three studies focused on the prevalence of female-specific endocrine conditions: one from 54 autistic women and 183 non-autistic women [38] and the other from 415 autistic women and 415 non-autistic women [39] recruited from the community, as well as another registry-based study on nation-wide electronic health records of 971 autistic women and 4855 non-autistic women [43]. The findings demonstrated higher rates of irregular menstrual cycle [38, 39], unusually painful periods [38, 39], and polycystic ovary syndrome (PCOS) [38, 43] in autistic women compared to non-autistic women, along with a range of other endocrine-related conditions [38, 39]. Furthermore, a nation-wide registries study of cancer risks with 8438 autistic children, youth, and young adults compared to 76,332 general population controls found a higher incidence of ovarian cancer in autistic girls/women compared to non-autistic girls/women [44]. Finally, a registry-based study on pregnancy outcomes in 2198 births to 1382 autistic women of child-bearing age, compared to 877,742 births in 503,846 women from the general population, found increased risk of preeclampsia, giving preterm birth, and receiving elective cesarean delivery for autistic women compared to non-autistic women [45].

### Miscellaneous emerging findings

Thirteen out of the 40 studies [46–58] were in emerging areas of research that did not align well with the three major themes; they also provided relatively inconsistent findings. All 13 studies offered information regarding autistic girls/women in comparison with autistic boys/men.

#### Additional neurological conditions

Three studies reported on neurological conditions beyond epilepsy in autism [46–48], with two examining neurological conditions as a broad category [46, 47] and the third, cerebral palsy [48]. One community-based study of 91 autistic children found that autistic girls had higher rates of neurological conditions than autistic boys [46]. A surveillance registry-based study found that in 6379 8-year-old autistic children across eight US sites, both autistic girls and boys showed stable rates of change in the prevalence of neurological conditions over 2002–2010 [47]. The final study, in a nation-wide cohort of 4180 autistic individuals (with ICD-10 Asperger’s syndrome), found increased prevalence of cerebral palsy in autistic individuals compared to the general population, without significant differences between autistic girls/women and boys/men [48].

#### Obesity and overweight

Two studies focused on obesity and overweight (via body mass index) [49, 50]. One registry-based study including 2976 autistic children and youth found that autistic girls were less likely to be obese compared to autistic boys, but they were equally likely to be overweight [49]. The other study, a community-based study of 78 autistic children, adolescents and adults, found that being overweight, as well as being obese, was more common in autistic men than in autistic women but with no differences found between autistic boys and autistic girls [50].

#### Gastrointestinal, metabolic, or nutritional problems

Four studies focused on gastrointestinal, metabolic, or nutritional conditions, comparing autistic girls/women and autistic boys/men [51–54]. One community-based study examined the prevalence of gastrointestinal problems in 169 autistic children, reporting that autistic girls had a greater likelihood of gastrointestinal problems than autistic boys and that more gastrointestinal symptoms were correlated with more severe core autistic symptoms [51]. Two were systematic reviews and meta-analyses: One, on peripheral iron levels and iron deficiency (1603 autistic children pooled from 25 studies across 3 analyses), found no sex/gender differences with respect to iron levels in autistic children [52]; the other, on mitochondrial dysfunction (648 autistic children and youth pooled from 65 studies), found a higher proportion of girls/women in the group of those with autism plus mitochondrial disease compared to those with autism only [53]. The final study, a community-based study on vitamins A and D deficiencies in 332 autistic children, reported that autistic girls had lower serum 25-OH vitamin D than autistic boys [54].

#### Immune profile

Three studies focused on sex-specific immunological features in autism [55–57]. Cytokine levels were measured in two studies of autistic children and adolescents from registry- and hospital-based cohorts: one with 144 (27 cytokines measured) [55] and the other, 87 (11 cytokines measured) [56] autistic individuals. Both studies found differences in the cytokine profiles of autistic girls compared to autistic boys but on different cytokines [55, 56]. A meta-analysis of 38 studies on 1393 autistic individuals regarding circulating pro-inflammatory cytokines also found sex/gender differences in cytokine patterns [57]. Altogether, these preliminary findings implicate potential sex/gender differences in immune profiles related to autism, though with inconsistent cytokine involvement.

#### Autism symptoms associated with physical health

Lastly, one meta-analysis examined sex/gender differences in 254 autistic adults regarding self-reported autistic characteristics, which included sensorimotor symptoms, some of which related to physical health, such as sensitivity to pain. Results revealed more severe sensorimotor symptoms in autistic women than in autistic men [58].

## Discussion

The purpose of this scoping review was to explore what is known about co-occurring physical health conditions in autistic girls/women. Out of the 201 full-text articles we reviewed, only 40 met our inclusion criteria, mainly due to the paucity of reporting on sex or gender differences among populations with autism and the low percentages of autistic girls/women included in the current literature. There is a pressing need for more research that includes large numbers of autistic girls/women in order to better understand their physical health. This should be prioritized in order to advance the best clinical care for autistic individuals [11].

Emerging patterns of co-occurring physical health conditions are worth further examination and replication. With respect to Theme 1, the current literature suggests that autistic girls/women tend to have more overall physical health challenges and lower overall health and quality of life than do autistic boys/men [19–31]. However, apart from epilepsy, it is still unclear which specific conditions are consistently more prevalent in autistic girls/women compared to autistic boys/men or to non-autistic girls/women [29–31]. Such inconsistency could be related to the substantial heterogeneity in the autism population even within each sex [59] and could also be related to confounding factors (e.g., genetic etiological load or other neurodevelopmental disabilities). Based on Theme 2, epilepsy is the most studied neurological condition in autism and has been relatively consistently reported to be more prevalent in autistic girls/women than in autistic boys/men [22, 33–35]. Furthermore, these studies highlight heightened autism symptoms, language impairment, motor dysfunction, older age, and presence of intellectual disability as potential confounding factors to epilepsy prevalence in autism [22, 33–35].

Findings in Themes 1 and 2 regarding differences between autistic girls/women and autistic boys/men need to be interpreted in light of the general pattern of case ascertainment in autism research so far, and the complexity associated with sex/gender-related differences in comorbidity pattern (e.g., association with intellectual disability) and etiological load. With regard to ascertainment bias, some have suggested that the observed association between autism and epilepsy is largely driven by co-occurring intellectual disability [60], which is in turn confounded by the greater proportion of autistic girls/women with lower cognitive abilities [61] and shared neurobiological bases between autism and neurological anomalies [62]. Indeed, for a long time, girls/women who were clinically diagnosed with autism, and hence included in the registry and hospital-based datasets in the reviewed studies, tended to be those with lower IQ, early-recognized and more “classic” symptoms of autism [63]—those who tend to carry heightened etiological risk factors for neurodevelopmental and physical health issues. Meanwhile, sex- and gender-related barriers to girls/women receiving an autism diagnosis [2, 4, 11] may result in autistic girls/women without evident intellectual disability or “classic” symptoms of autism being under-represented in the current literature. Therefore, the observed differences between autistic boys/men and autistic girls/women in overall physical health status and epilepsy are likely confounded by sex- and gender-based ascertainment bias. It is also likely that the later recognition of autism in some girls/women has resulted in inadequate health care [64–66], further contributing to poorer overall health status. However, it remains unclear if the same pattern of male–female differences holds in autistic individuals who are so far under-recognized and undiagnosed.

At the same time, these observed sex/gender differences could be associated with variations in etiological load. At a group level, autistic girls/women tend to carry more de novo protein-truncating genetic variants likely causal to autism compared to autistic boys/men [67]. Given the pleiotropic effects of many autism-related genes, autistic individuals who carry these variants are more likely to experience other neurodevelopmental (e.g., intellectual disability) and physical health challenges (e.g., epilepsy, other neurological anomalies, cardiovascular defects, obesity) [8]. This means that the observed sex/gender differences may further reflect, and are confounded by, higher co-occurrence of neurodevelopmental disabilities and stronger de novo genetic etiological load in diagnosed autistic girls/women compared to autistic boys/men.

Another pattern that emerged (Theme 3) was the greater burden of co-occurring endocrine or reproductive health concerns in autistic girls/women (e.g., menstruation-related challenges, hormonal conditions, and ovarian cancer). Nevertheless, these findings should be viewed as preliminary, owing to the moderate sample sizes [40–42] and the reliance on self-report questionnaires—rather than direct clinical assessments—to characterize female-specific endocrine conditions [38–42]. Interestingly though, this theme can be hypothesis generating with implications for plausible biological mechanisms underlying autism, endocrine and other associated biological alternations. Some have speculated that endocrine dysregulation in autistic girls/women is partly indicative of altered prenatal sex steroid exposure [68, 69], which may contribute to both endocrine dysregulation and autism-related neurodevelopmental and behavioral characteristics [70–72]; see recent emerging empirical support [73–77]. How such prenatal endocrine factors contribute to the mechanisms leading to autism and sex differences in concurrent physical health disorders remains unclear and is an area requiring more in-depth mechanistic investigation. There is growing evidence supporting the role of multidirectional interactions between hormonal regulation, prenatal immune activation, epigenetic regulation in key brain regions, and postnatal environments in producing a range of distinct but related autistic phenotypes [78–80]. It is possible that there are shared mechanisms underlying autism and co-occurring endocrine and immune alterations, with sex differential mechanisms involved.

Finally, there is increasing evidence that gastrointestinal [81] and metabolic/nutritional conditions [82], including obesity and diabetes [83], have a high frequency of co-occurrence in autistic individuals. These conditions could involve shared etiological mechanisms with autism as well as with life experiences (e.g., lifestyle, health care support, medication use) of autistic people. The emerging but preliminary findings regarding sex/gender differences in these domains suggest that they may be especially important to the physical health of autistic girls/women. Elucidating associated sex/gender-related mechanisms requires more in-depth research.

### Clinical Implications (also see Fig. 2)

**Fig. 2:**
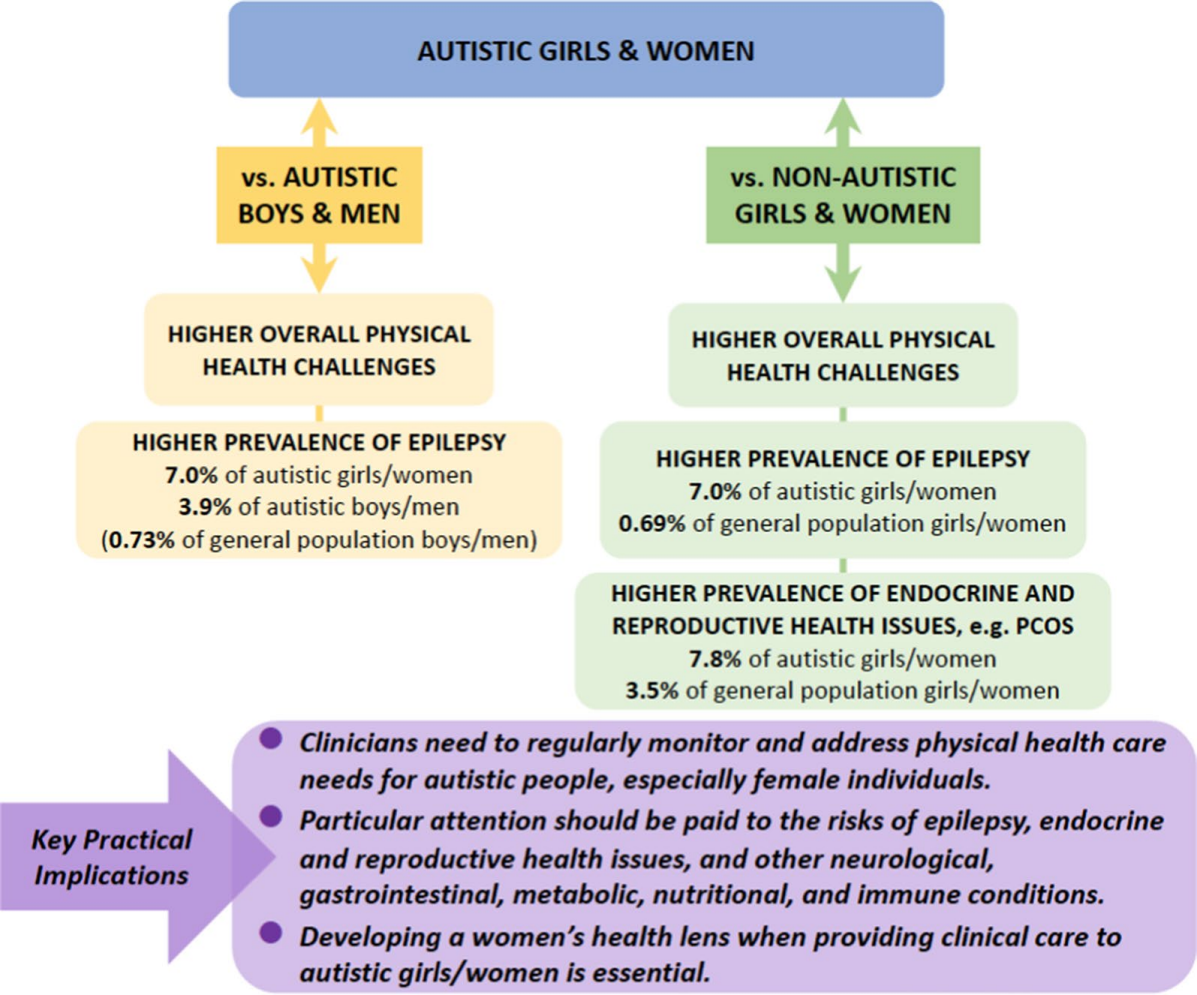
Exemplary findings and clinical implications. Prevalence rates of exemplary physical health conditions were taken from the largest sample size studies included in this review (i.e., [43] for polycystic ovary syndrome [PCOS] and [35] for epilepsy) and a representative study in the general population (i.e., for epilepsy [93])

The finding that autistic girls/women experience more overall physical health challenges than non-autistic girls/women and autistic boys/men is of immediate clinical relevance. Improving physical health is integral to the care of all autistic individuals [84, 85]. Frontline and primary care clinicians should regularly monitor and address health care needs for autistic children, youth, and adults alike [86, 87], especially for female individuals [88]. For autistic girls/women, this scoping review provides indication for clinicians to particularly attend to the risks of epilepsy, endocrine and reproductive health issues, as well as other neurological, gastrointestinal, metabolic, nutritional, and immune conditions, among various physical health issues. To achieve holistic support, developing a women’s health lens when providing clinical care to autistic girls/women is essential and will significantly enrich sex- and gender-informed health care for all autistic people. Sex- and gender-informed health promotion strategies need to be applied across the life span. Conversely, improved attention to physical health in girls/women who also experience difficulties in social communication, restricted/stereotyped behaviors and sensory issues might also facilitate the identification of later recognized autism in girls/women [89].

Another key consideration is the interplay between physical and mental health. Autistic people are prone to experiencing mental health challenges (which we did not review here) [15]. However, many psychiatric medications for such challenges have side effects that are more commonly experienced in autistic compared to non-autistic individuals [84, 90, 91], contributing to heightened risk to physical health (e.g., weight gain and endocrine problems related to psychotropic medications). These findings have not yet been sufficiently studied in a sex-/gender-specific manner. Meanwhile, physical health challenges (e.g., epilepsy, hormonal dysregulation) can have detrimental impacts on mental health and affect mood and behavior. Such complexity and interplay may result in the high clinical needs and multiple service use that are common in the autism population, particularly among girls/women [64–66]. Many of these specific physical health challenges are treatable with the proviso that clinical trials need to disaggregate their data by sex and gender, which is unfortunately still insufficiently done for trials involving autistic people.

Timely diagnosis and treatment will enhance well-being associated with both physical and mental health of autistic individuals across the life span. This review has revealed that autistic girls/women are a population with unique health needs. Therefore, it requires us to develop comprehensive services that integrate developmental, mental, and physical health for autistic girls/women.

## Limitations

There are several limitations to consider in interpreting our findings. First, it is possible that we were unable to identify all studies relevant to our guiding questions due to the heterogeneity in how physical health conditions were assessed and reported in the literature. Nevertheless, based on the principles of a scoping review, we have identified potential areas in the literature that warrant future investigation and areas with insufficient information as yet to make firm conclusions. Second, the decision to focus on physical health, thus excluding studies only focusing on psychiatric co-occurring conditions, meant that we could not explore how mental health and physical health are intertwined in autistic people, particularly in girls/women. Finally, our scoping review demonstrates that understandings of the physical health of autistic girls/women are still emerging. The limited number of studies in each theme, and their varying quality and research methodologies, makes it difficult to reach definitive conclusions.

However, several lessons about significant gaps in the clinical and research literature about autism can be learned from our review to inform future research directions. There is a lack of consistent, basic epidemiological information on the prevalence and incidence for co-occurring physical health conditions in the autism population by sex and, in particular, by gender. Reliable and valid measurement tools for physical health in autistic individuals need to be further developed. Additionally, there are insufficient longitudinal studies to chart the emergence of co-occurring conditions and randomized control trials to assess treatment for these conditions. There are also insufficient biological studies on the mechanisms of the development of physical health challenges in autism by sex and by gender. Further, there is significant underrepresentation of autistic girls/women in most studies, and only a small minority of studies are equipped to or have formally examined and reported sex/gender differences in their primary analyses. These highlight the ignorance about how sex and gender influence autism, perhaps due to a male-biased lens. The relative lack of awareness about women’s health and female experiences in the scientific and clinical knowledge about physical health and autism leaves girls/women diagnosed with autism at distinct disadvantages. Targeted research on autistic girls/women is clearly needed. Future studies on autism should be designed to achieve sex/gender equity by ensuring a male/female ratio closer to the general population rate (i.e., ~ 3:1) [4]. For research focusing on delineating sex-related or gender-related effects, balanced inclusion of participants with diverse sexes and genders should be targeted [2, 92]. Finally, it is extremely rare in the current empirical literature that sex (biological attributes) and gender (psychological, social, and cultural attributes) are defined and examined separately and in valid ways. These gaps need to be addressed in future research, alongside a clarification of sources of demographic, clinical, and etiological heterogeneity such as age, pubertal stage, developmental trajectories, intellectual functioning, and genetic background. Such clarification is fundamental for future studies to generate etiological and mechanistic insights by studying co-occurring physical health conditions in autism using sex- and gender-informed frameworks.

## Conclusions

To our knowledge, this is the first scoping review on physical health in autistic girls/women. Emerging themes suggest that autistic girls/women tend to have heightened rates of a variety of co-occurring physical health challenges compared to autistic boys/men and non-autistic girls/women. Clinicians should provide holistic care that integrates not only developmental and mental health, but also physical health. Future studies need to include sufficient numbers of autistic girls/women to achieve adequate power, attend to physical health and the intertwined nature of developmental, mental, and physical health, and use sex- and gender-informed lenses.

## Supplementary information

**Additional file 1.** Appendix: Search Strategy.

## Data Availability

Not applicable.

## Abbreviations

DSM: Diagnostic and Statistical Manual of Mental Disorders
ICD: International Classification of Diseases
OR: Odds ratio
PRISMA: Preferred Reporting Items for Systematic Reviews and Meta-analyses
RR: Risk ratio
SD: Standard deviation
SIR: Standardized incidence ratio
