# Supplementary material for: Physical health of autistic girls and women: a scoping review
Source: Mol Autism. 2020 Oct 27;11:84. doi: 10.1186/s13229-020-00380-z (PMC7590704; doi:10.1186/s13229-020-00380-z)
Supplement: Supplementary file 1 — Additional file 1. Appendix: Search Strategy. [file 13229_2020_380_MOESM1_ESM.docx]

**Appendix: Search Strategy**

**Concepts / keywords:**

1. Female (MeSH terms)

women

female*

sex differences

1. Autism spectrum disorders (MeSH major topic)

autistic disorder (MeSH terms)

autism

pervasive developmental disorder

autism spectrum conditions

1. Co-morbid health conditions

co-occurring

co-morbidity (MeSH terms)

comorbidity

comorbid

concomitant

co-existing

co-existence

health conditions

health issues

health impairment

Health (MeSH terms) and “problems”

physical impairment

physical health

other medical conditions

other chronic disease(s)

multimorbidity

multi-morbidity

multiple disease(s)

medical conditions

associated disorder(s)

associated disease(s)

***PubMed***

(((((female[Title/Abstract]) OR females[Title/Abstract]) OR women[Title/Abstract]) OR "sex differences"[Title/Abstract])) AND  ((((((comorbidity[Title/Abstract] OR co-morbidity[Title/Abstract] OR comorbid[Title/Abstract] OR “other medical conditions”[Title/Abstract] OR “other chronic disease*”[Title/Abstract] OR multimorbidity[Title/Abstract] OR multi-morbidity[Title/Abstract] OR multiple disease*[Title/Abstract] OR multiple morbid*[Title/Abstract] OR polypathology[Title/Abstract] OR associated disease*[Title/Abstract] OR associated disorder*[Title/Abstract] OR co-existence[Title/Abstract] OR co-existing[Title/Abstract] OR concomitant[Title/Abstract] OR co-occurring[Title/Abstract] OR "health conditions"[Title/Abstract] OR "health problems"[Title/Abstract] OR "health issues" [Title/Abstract]))) AND (((((autism spectrum disorder[MeSH Terms]) OR autism spectrum disorders[MeSH Terms]) OR autistic disorder[MeSH Terms]) OR pervasive development disorder[MeSH Terms]) OR pervasive development disorders[MeSH Terms]))

***Scopus***

( ( TITLE-ABS-KEY ( autism  OR  autistic  AND disorder  OR  autistic  AND disorders  OR  autism  AND spectrum  AND disorder  OR  autism  AND spectrum  AND disorders  OR  pervasive  AND developmental  AND disorder  OR  pervasive  AND developmental  AND disorders ) )  AND  ( ( TITLE-ABS ( comorbidity  OR  co-morbidity  OR  comorbid  OR  "other medical conditions"  OR  "other chronic disease*"  OR  multimorbidity  OR  multi-morbidity  OR  multiple  AND disease*  OR  multiple  AND morbid*  OR  polypathology  OR  associated  AND disease*  OR  associated  AND disorder*  OR  co-existence ) )  OR  ( TITLE-ABS-KEY ( co-existing  OR  concomitant  OR  co-occurring  OR  ( health  AND  conditions )  OR  ( health  AND  problems )  OR  ( health  AND  issues ) ) ) ) )  AND  ( TITLE-ABS ( female  OR  females  OR  women  OR  ( sex  AND  differences ) ) )

***CINAHL***

(MH "Autistic Disorder") OR (MH "Pervasive Developmental Disorder-Not Otherwise Specified") OR (MH "Asperger Syndrome") AND TX female OR females OR women OR "sex differences" AND TI comorbidity or co-morbidity or comorbid or "other medical conditions" or "other chronic disease" or "multimorbidity" or "multi-morbidity" or "multiple disease*" or "multiple morbid*" or polypathology or "associated disease*" or "associated disorder*" or co-existence or co-existing or concomitant or co-occurring or "health conditions" or "health problems" or "health issues" or "health status"

***PsycInfo***

exp Autism Spectrum Disorders/ AND ( (comorbidity or co-morbidity or comorbid or "other medical conditions" or "other chronic disease" or "multimorbidity" or "multi-morbidity" or "multiple disease*" or "multiple morbid*" or polypathology or "associated disease*" or "associated disorder*" or co-existence or co-existing or concomitant or co-occurring or "health conditions" or "health problems" or "health issues" or "health status").mp. [mp=title, abstract, heading word, table of contents, key concepts, original title, tests & measures]) AND (female or females or women or "sex differences").mp. [mp=title, abstract, heading word, table of contents, key concepts, original title, tests & measures]

***EMBASE***

(female or females or women or "sex differences").mp. [mp=title, abstract, heading word, drug trade name, original title, device manufacturer, drug manufacturer, device trade name, keyword, floating subheading word] AND exp autism/ AND (comorbidity or co-morbidity or comorbid or "other medical conditions" or "other chronic disease" or "multimorbidity" or "multi-morbidity" or "multiple disease*" or "multiple morbid*" or polypathology or "associated disease*" or "associated disorder*" or co-existence or co-existing or concomitant or co-occurring or "health conditions" or "health problems" or "health issues" or "health status").m_titl.
